# Normal Early Investigations Do Not Exclude Bickerstaff Brainstem Encephalitis: Highlighting Diagnostic and Treatment Pitfalls

**DOI:** 10.1155/crnm/8222960

**Published:** 2026-10-26

**Authors:** Hidaya Kmail, Mohammad Jaradat, Ahmad Abbas, Omar Abu Zaineh, Alaa Kharouf, Qasem N. AlShaer

**Affiliations:** ^1^ Faculty of Medicine Arab American University (AAUP) Jenin State of Palestine aauj.edu; ^2^ Department of Internal Medicine Ibn Sina Hospital Jenin State of Palestine ibnsinatrust.com

**Keywords:** antiganglioside antibodies, Bickerstaff brainstem encephalitis, F-waves, Guillain–Barré syndrome, plasma exchange

## Abstract

Bickerstaff brainstem encephalitis (BBE) is a rare immune‐mediated disorder characterized by ophthalmoplegia, cerebellar ataxia, and impaired consciousness. It is now recognized as a variant of Guillain–Barré syndrome (GBS), sharing common pathogenic mechanisms with Miller Fisher syndrome. However, early diagnosis remains challenging, particularly when initial investigations are normal. We report a 20‐year‐old previously healthy woman presenting with rapidly progressive ophthalmoplegia, bulbar dysfunction, flaccid quadriparesis, areflexia, and impaired consciousness following a febrile upper respiratory illness. Early cerebrospinal fluid analysis and brain MRI were normal. Anti‐GQ1b antibodies were positive, and nerve conduction studies showed isolated proximal conduction abnormalities with absent F‐waves. The patient developed respiratory failure requiring mechanical ventilation and was treated with an extended course of plasma exchange, resulting in near‐complete recovery. This case highlights a critical diagnostic pitfall: BBE, a variant of GBS, can present with a life‐threatening brainstem syndrome despite entirely normal early investigations. We emphasize the value of recognizing the paradoxical combination of areflexia and extensor plantar responses and the use of F‐wave studies as an early electrophysiological foothold when standard studies are unrevealing. Furthermore, it demonstrates that even in catastrophic, ventilator‐dependent presentations, an extended course of plasma exchange can lead to a favorable outcome, suggesting a potential therapeutic strategy for severe presentations of BBE.

## 1. Introduction

Bickerstaff brainstem encephalitis (BBE) is a rare postinfectious neurological disorder characterized by ophthalmoplegia, cerebellar ataxia, and altered consciousness, reflecting combined central and peripheral nervous system involvement [1]. It is now considered part of a continuous spectrum of immune‐mediated neuropathies that includes Guillain–Barré syndrome (GBS) and its variants, most notably Miller Fisher syndrome (MFS) [2]. These disorders share common features, most notably the presence of antiganglioside antibodies, particularly anti‐GQ1b [3]. BBE is distinguished from its counterparts by central nervous system signs, impaired consciousness, hyperreflexia in later stages, or extensor plantar responses, superimposed on the peripheral neuropathy typical of GBS [1].

Severe presentations of BBE with prominent peripheral involvement are uncommon but clinically significant, as they often present with severe weakness, bulbar involvement, and respiratory failure [4]. Early diagnosis is challenging due to variable imaging findings, initially normal cerebrospinal fluid (CSF) analysis, and evolving electrophysiological features [3, 5, 6]. We present a case of BBE with normal early investigations to emphasize these diagnostic challenges, the value of subtle early findings, and the potential for excellent recovery with prompt, aggressive immunotherapy.

## 2. Case Presentation

A healthy 20‐year‐old Middle Eastern female presented with rapidly progressive weakness of two days’ duration. She first noticed difficulty walking; her legs felt heavy, and she required support to maintain balance. Within hours, she could no longer stand unassisted. Her family, who provided the history due to her dysarthria, described a febrile upper respiratory illness one week prior: sore throat, nasal congestion, and low‐grade fever that resolved with antipyretics. Over the 24 h preceding admission, she developed blurred vision, frontal headache, dysarthria, and dysphagia. Lumbar puncture at the referring hospital was reportedly normal.

By the time of transfer to our tertiary center, neurological examination revealed a striking constellation of findings (Table 1). The patient was drowsy, with both eyes closed due to bilateral ptosis. She was in distress, with severe weakness limiting full mental status assessment, though she inconsistently followed simple commands. Cranial nerve examination demonstrated near‐complete bilateral ptosis and complete ophthalmoplegia with limitation of gaze in all directions, not corrected by vestibulo‐ocular reflex. Pupils were equal and reactive. She had severe bulbar weakness resulting in inability to communicate and pooling of secretions.

**TABLE 1:** Summary of neurological findings during hospitalization.

Domain	At presentation	ICU course	Predischarge
Mental status	Drowsy, following simple commands, but not complex ones; speech severely dysarthric	Gradual improvement in consciousness and interaction	Alert, oriented, intact memory, and higher functions
Cranial nerves	Bilateral ptosis, marked ophthalmoplegia, facial weakness, dysarthria	Persistent ophthalmoplegia with gradual bulbar improvement	Significant recovery of bulbar and ocular function
Motor power	Generalized flaccid quadriparesis	Progressive improvement with physiotherapy and PLEX	5/5, able to ambulate
Tone	Flaccid	Improving tone	Tone normalized
Reflexes	Areflexia in all limbs, right Babinski present	Gradual return of reflexes	Hyperreflexia
Sensory exam	unable to assess	Intact	Intact
Respiratory involvement	Respiratory muscle weakness requiring intubation	Extubated after clinical improvement	Breathing comfortably on room air
Coordination/gait	Unable to walk due to severe weakness and imbalance	Able to stand briefly with support	Able to walk independently

Motor examination showed flaccid tone throughout. Neck flexor strength was reduced. Upper extremity power was asymmetrical: the right upper extremity showed weakness of variable muscles in the 3‐4/5 range, while the left was stronger. In the lower extremities, the patient could only withdraw to painful stimuli. Deep tendon reflexes were absent in all four extremities. Surprisingly, Babinski’s sign was present on the right, a paradoxical combination of peripheral areflexia and a central deficit. There was no sensory level or evidence of meningeal irritation, and the general physical examination was, otherwise, unremarkable. Given the depressed mental status, bulbar weakness with inability to handle secretions, and severe weakness, endotracheal intubation was performed, and the patient was admitted to the intensive care unit.

Initial CSF analysis at the referring hospital on Day 0 showed protein 31 mg/dL, white blood cells 0 cells/µL, and glucose 61 mg/dL. A repeat CSF study on Day 2 after admission, including more extensive testing, demonstrated protein 36 mg/dL, WBC 0 cells/µL, and RBC 0 cells/µL, with negative cytology and flow cytometry. IgG index was 0.5 (normal < 0.7) (Table 2). CSF and serum viral panels, including West Nile virus IgM, were negative.

**TABLE 2:** CSF analysis.

Name	Result: Day 2	Result: Day 0	Normal range
Appearance	Clear/transparent	Clear/transparent	Clear
Glucose in fluid	65 mg/dL	61 mg/dL	40–76 mg/dL
Glucose in serum	110 mg/dL		70–150 mg/dL
Glucose ratio (fluid/serum)	0.59	0.6	0.6
RBCs	No cell seen	No cell seen	0–5 Cell/mm
WBCs	No cell seen	No cell seen	0–4 Cell/mm
Total protein in fluid	35.8 mg/dL	31.4 mg/dL	0–45 mg/dL
IgG index	0.5		< 0.7

Serological workup revealed positive serum anti‐GQ1b antibodies. Antiacetylcholine receptor and anti‐MuSK antibodies were negative. Autoimmune screening including ANA and ANCA was negative. Brain magnetic resonance imaging with contrast showed no abnormalities in the brainstem or cerebellum (Figure 1). EEG showed normal background with no epileptiform activity.

**FIGURE 1:**
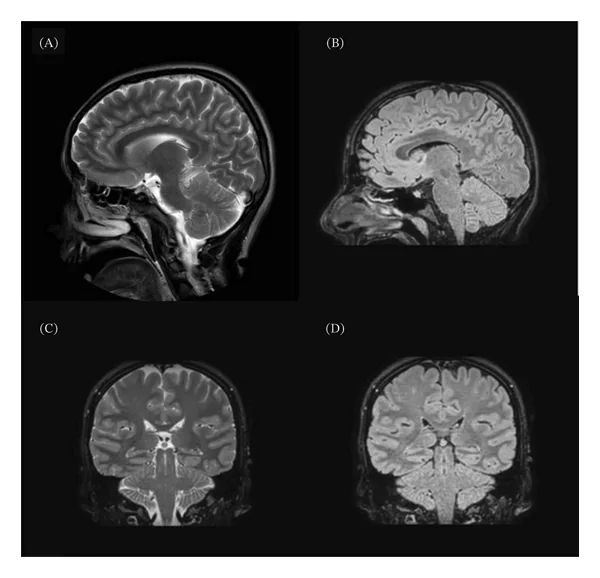
Brain magnetic resonance imaging demonstrating no structural abnormalities. (A) Sagittal T2‐weighted image. (B) Sagittal FLAIR image. (C) Coronal T2‐weighted image. (D) Coronal FLAIR image. No signal abnormalities were identified in the brainstem or cerebellum.

Nerve conduction study performed on Day 5, while the patient was intubated, revealed normal motor amplitudes, distal latencies, and conduction velocities in the arms and legs. The only abnormality was absent or inconsistent F‐waves, suggesting demyelination of proximal nerve segments (Figure 2A). Repetitive nerve stimulation showed no decrements.

**FIGURE 2:**
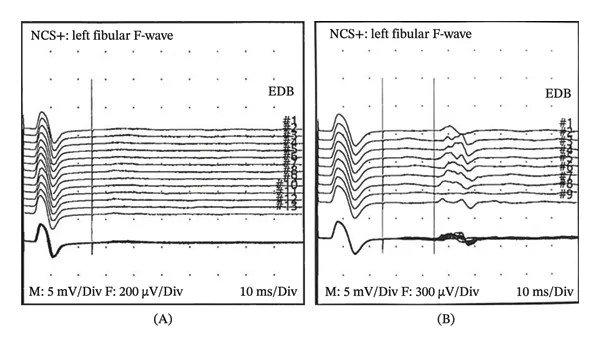
Transient F‐wave abnormality in the left fibular nerve. (A) Nerve conduction study at admission demonstrating absent F‐wave responses following repeated stimulation of the left fibular nerve. (B) Repeat study prior to discharge showing restoration of reproducible F‐wave responses, consistent with resolution of proximal conduction dysfunction.

Based on the presence of ophthalmoplegia, bulbar dysfunction, impaired consciousness, flaccid quadriparesis, areflexia with upgoing plantar reflex, antecedent infection, and positive anti‐GQ1b antibodies, a diagnosis of BBE, a GBS variant within the anti‐GQ1b antibody spectrum, was established. Acute myasthenia gravis crisis was initially suspected but deemed unlikely given the depressed mental status despite normal arterial blood gases (no CO_2_ retention) and the combination of areflexia with upgoing toes. Acute flaccid paralysis was considered, but upper motor signs and negative CSF and viral studies made this unlikely.

The initial gait impairment and inability to maintain balance were interpreted as predominantly cerebellar ataxia in the context of BBE, rather than sensory ataxia or impairment attributable solely to motor weakness. Formal coordination testing was limited by the severity of the patient’s quadriparesis and impaired consciousness.

The patient was admitted to the ICU and remained intubated. Therapeutic plasma exchange (PLEX) was initiated on the same day of admission to our hospital (Day 2 of symptom onset). She received a total of 8 sessions over 19 days, exchanging 1–1.5 plasma volumes per session with 5% human albumin as the replacement fluid every other day. This extended course was guided by the severity of her presentation and the gradual nature of her recovery. Her course was complicated by multiple transient issues, all of which resolved with proper management.

Gradual neurological improvement was observed following PLEX, first noticed in the second week. The patient was successfully extubated after 13 days. She received intensive physiotherapy, and muscle strength improved progressively. During the second week, while still intubated, deep tendon reflexes returned, and she even developed transient hyperreflexia with increased tone. Bulbar symptoms resolved, and a modified barium swallow study demonstrated no aspiration. Repeat nerve conduction study on Day 21 showed normalization of all parameters, with distal amplitudes, latencies, and conduction velocities remaining within normal limits and no delayed peripheral demyelinating features; the only change from the initial study was resolution of the previously noted abnormality with reemergence of F‐waves (Figure 2B).

At discharge, she was fully conscious, ambulatory, and had near‐normal muscle strength. On follow‐up in the outpatient clinic, her ptosis resolved, and eye movements were normal without diplopia. Strength was 5/5 in all tested muscle groups, and she was walking independently without assistance. A timeline of clinical course from prodromal symptoms through intensive care management and recovery is summarized in Figure 3.

**FIGURE 3:**
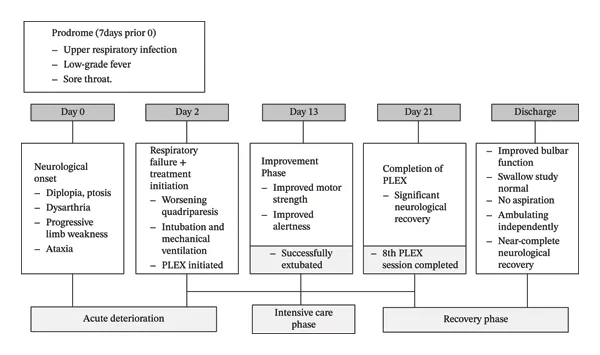
Timeline illustrating the patient’s clinical progression from prodromal symptoms through neurological deterioration, intensive care management with plasma exchange, and subsequent recovery.

## 3. Discussion

This case of BBE illustrates several critical points for clinicians. The most immediate challenge was the diagnostic dilemma posed by completely normal early investigations. Our patient’s CSF was acellular with normal protein on two separate occasions during the first week, and her brain MRI was unremarkable. Albuminocytologic dissociation, a hallmark of GBS, may be absent in up to 50% of the patients during the first week and is even less consistent in BBE [7]. Similarly, brain imaging in BBE is frequently normal, with abnormalities reported in only a minority of cases [1]. This pattern, a rapidly progressive, life‐threatening brainstem syndrome with a normal initial workup, can easily lead to diagnostic delay. Our case demonstrates that BBE must remain on the differential diagnosis even when early investigations are entirely unrevealing.

In this diagnostic vacuum, two findings were pivotal. First, the clinical examination itself provided the most important clue. The simultaneous presence of areflexia and an extensor plantar response is a paradoxical combination that is a hallmark of the anti‐GQ1b antibody spectrum and should immediately raise suspicion for BBE [4]. Second, the early nerve conduction study, while, otherwise, normal, revealed isolated, absent F‐waves. F‐waves assess the proximal nerve segment, including the nerve root. Their absence with preserved distal responses suggests early proximal involvement due to conduction failure at the nerve roots, before distal demyelination is established [8]. This transient F‐wave abnormality, which resolved with clinical improvement, is an under‐recognized early electrophysiological clue that can provide crucial diagnostic support when other studies are normal.

The lateralization of the extensor plantar response to the right side, corresponding to the side of greater upper extremity weakness, raises the question of whether these findings share a common pyramidal origin. However, we believe this concordance is coincidental rather than mechanistic. The upper extremity weakness in this case was flaccid and accompanied by generalized areflexia, a pattern consistent with the peripheral demyelinating/axonal process of the GBS‐spectrum polyradiculoneuropathy rather than an upper motor neuron lesion. The Babinski sign, in contrast, reflects central nervous system involvement characteristic of BBE. The co‐occurrence of a peripheral pattern of weakness with a central pyramidal sign in the same limb is not unexpected in BBE‐spectrum disease, where central and peripheral processes coexist, and does not imply a shared focal lesion.

The therapeutic impact of our approach was significant. The patient received eight sessions of PLEX over 19 days, an extended course compared to the standard 4‐5 exchanges. This decision was guided by the severity of her presentation and a slow initial response. While high‐quality evidence for extended PLEX in severe GBS/BBE is lacking, expert opinion supports its consideration in patients with a catastrophic presentation or poor early response [9]. In rare case examples reported in the literature, IVIG treatment administered during the early diagnosis period, within the first few days, has been reported to be effective [10]. Our patient’s near‐complete recovery after this extended regimen suggests it may be a beneficial strategy in severe BBE presentations, a point for future study.

Our patient’s positive anti‐GQ1b antibodies place her within the spectrum of disorders associated with molecular mimicry following infection [9]. *Campylobacter jejuni* and *Haemophilus influenzae* are the best‐characterized triggers, though many upper respiratory pathogens can precipitate the autoimmune response [11]. Bacterial infections beyond these classic triggers have also been reported to precede BBE, underscoring the breadth of antecedent pathogens that can incite this antibody response [12]. Anti‐GQ1b antibodies bind to ganglioside GQ1b, which is highly expressed in oculomotor nerves, muscle spindles, and the brainstem reticular formation [13].

Although our patient was an adult, her clinical course parallels phenotypes described in pediatric and young‐onset BBE cohorts, where rapid symptom progression and initially unremarkable CSF and MRI findings are common, yet full neurological recovery remains achievable with timely immunotherapy [6, 14]. This case offers a unique contribution to the literature. While large series from Odaka et al. [1] and Giaccari et al. [6] have characterized the clinical features of BBE, they do not specifically highlight the triad of (1) a completely normal initial workup (CSF and MRI), (2) isolated F‐wave abnormalities as an early diagnostic foothold, and (3) the successful use of an extended PLEX protocol in a severe, ventilator‐dependent patient. To our knowledge, this is among the first reports to integrate all three elements, providing a practical roadmap for clinicians facing a similar challenge.

In conclusion, BBE represents a severe form of autoimmune neurologic disease. This case demonstrates that even in the complete absence of early laboratory or imaging support, recognizing the clinical syndrome, particularly the coexistence of areflexia and an extensor plantar response should prompt immediate immunotherapy. Early electrophysiology may reveal subtle but valuable clues like absent F‐waves. Despite a catastrophic presentation, our patient achieved complete neurological recovery following early and extended plasma exchange, underscoring that prompt, aggressive management can lead to a favorable prognosis.

## Author Contributions

Hidaya Kmail: investigation, data curation, and writing–original draft. Mohammad Jaradat: investigation and data curation. Ahmad Abbas: investigation and data curation. Omar Abu Zaineh: resources, investigation, and writing–review and editing. Alaa Kharouf: resources and investigation. Qasem N. AlShaer: supervision, investigation, and writing–review and editing.

## Funding

No funding was received for this case report.

## Disclosure

All authors have read and approved the final manuscript.

## Ethics Statement

This report describes routine clinical care of a single patient. Formal Institutional Review Board/Ethics Committee approval was not required for publication of this case report, in accordance with institutional policy for single‐patient case reports.

## Consent

Written informed consent for publication of this case report and any accompanying images was obtained from the patient.

## Conflicts of Interest

The authors declare no conflicts of interest.

## Supporting Information

Additional supporting information can be found online in the Supporting Information section.

## Supporting information


**Supporting Information** CARE‐checklist BBE.

## Data Availability

The data that support the findings of this study are available from the corresponding author upon reasonable request.
