# Supplementary material for: Normal Early Investigations Do Not Exclude Bickerstaff Brainstem Encephalitis: Highlighting Diagnostic and Treatment Pitfalls
Source: Case Rep Neurol Med. 2026 Oct 26;2026:8222960. doi: 10.1155/crnm/8222960 (PMC13698646; doi:10.1155/crnm/8222960)
Supplement: Supplementary file 1 [file CRNM-2026-8222960-s001.docx]

CARE Checklist of information to include when writing a case report

**Topic	Item	Checklist item description	Reported on Line**

**Title	1**	The diagnosis or intervention of primary focus followed by the words “case report” . . . . . . . . . . . . . . . . . .	Yes, Line 1-2

**Key Words	2**	2 to 5 key words that identify diagnoses or interventions in this case report, including "case report"	Yes, Lines 38-39

**Abstract**

**(no references)**

**3a**	Introduction: What is unique about this case and what does it add to the scientific literature?	Yes, Lines 20-23

**3b**	Main symptoms and/or important clinical findings . . . . . . . . . . . . . . . . . . . . . . . . . . . . . . . . . . . . . . . . . . . . . . . . . . .	Yes, Lines 24-26

**3c**	The main diagnoses, therapeutic interventions, and outcomes	Yes, Lines 26-30

**3d**	Conclusion—What is the main “take-away” lesson(s) from this case?	Yes, Lines 31-37

**Introduction	4**	One or two paragraphs summarizing why this case is unique (**may include references**)	Yes, Lines 42-56

**Patient Information**

**5a**	De-identified patient specific information	Yes, Line 58

**5b**	Primary concerns and symptoms of the patient	Yes, Lines 59-64

**5c**	Medical, family, and psycho-social history including relevant genetic information	Yes, Line	62

**5d**	Relevant past interventions with outcomes	Yes, Line 64

**Clinical Findings**

**Timeline**

**Diagnostic Assessment**

**Therapeutic Intervention**

**Follow-up and Outcomes**

6 Describe significant physical examination (PE) and important clinical findings	Yes, Lines 65-80
7 Historical and current information from this episode of care organized as a timeline	Yes, Figure 3

**8a**	Diagnostic testing (such as PE, laboratory testing, imaging, surveys).	Yes, Lines 82-95

**8b**	Diagnostic challenges (such as access to testing, financial, or cultural)	N/A

**8c**	Diagnosis (including other diagnoses considered)	Yes, Lines 97-104

**8d**	Prognosis (such as staging in oncology) where applicable	N/A

**9a**	Types of therapeutic intervention (such as pharmacologic, surgical, preventive, self-care) . . . . . . . . . . . . . . . . .	Yes, Lines 106-108

**9b**	Administration of therapeutic intervention (such as dosage, strength, duration)	Yes, 106-108

**9c**	Changes in therapeutic intervention (with rationale)	N/A

**10a**	Clinician and patient-assessed outcomes (if available)	Yes, Lines 112-122

**10b**	Important follow-up diagnostic and other test results	Yes, Lines 116-118

**10c**	Intervention adherence and tolerability (How was this assessed?)	Yes, 107-109

**10d**	Adverse and unanticipated events	Yes, Lines 109-110

**Discussion	11a**	A scientific discussion of the strengths AND limitations associated with this case report	Yes, Lines 124-163

**11b**	Discussion of the relevant medical literature **with references**	Yes, Lines 157-163

**11c**	The scientific rationale for any conclusions (including assessment of possible causes)	Yes, Lines 124-163

**11d**	The primary “take-away” lessons of this case report (without references) in a one paragraph conclusion	Yes, Lines 165-172

**Patient Perspective	12**	The patient should share their perspective in one to two paragraphs on the treatment(s) they received . . . .	No, Patient was unavailable for follow-up interview to share her perspective at the time of manuscript preparation.

**Informed Consent	13**	Did the patient give informed consent? Please provide if requested . . . . Yes, Lines 174-175	**Yes** **No**
